# Effects of peer education intervention on HIV/AIDS related sexual behaviors of secondary school students in Addis Ababa, Ethiopia: a quasi-experimental study

**DOI:** 10.1186/s12978-015-0077-9

**Published:** 2015-09-07

**Authors:** Takele Menna, Ahmed Ali, Alemayehu Worku

**Affiliations:** St Paul’s Hospital Millennium Medical College, P.O.Box:1271, Addis Ababa, Ethiopia; School of Public Health, College of Health Sciences, Addis Ababa University, P.O. Box: 33412, Addis Ababa, Ethiopia

## Abstract

**Background:**

Worldwide, about 50 % of all new cases of HIV occur in youth between age 15 and 24 years. Studies in various sub-Saharan African countries show that both out of school and in school adolescents and youth are engaged in risky sexual behaviors.

School-based health education has been a cornerstone of youth-focused HIV prevention efforts since the early 1990s. In addition, peer-based interventions have become a common method to effect important health-related behavior changes and address the HIV/AIDS pandemic. Thus, the aim of this study was to evaluate efficacy of peer education on changing HIV related risky sexual behaviors among school youth in Addis Ababa, Ethiopia.

**Methods:**

A quasi experimental study with peer education intervention was conducted in purposively selected four secondary schools (two secondary schools for the intervention and other two for the control group) in Addis Ababa, Ethiopia. Five hundred sixty students from randomly selected sections of grade 11 were assessed through anonymous questionnaires conducted in pre- and post-intervention periods. Pertinent data on socio-demographic and sexual behavior related factors were collected. The statistical packages used for data entry and analysis were epi-info version 3.5.4 and SPSS version 20.0 respectively. Chi-square test and multivariable logistic regressions were used for testing association between peer education intervention and sexual behaviors of students. In addition to testing association between dependent and independent variables, multi-variable analysis was employed to control for the effects of confounding variables.

**Results:**

When the pre and post intervention data of each group were compared, comprehensive Knowledge of HIV (*P*-Values =0.004) and willingness to go for HIV counseling and testing (*P*-value = 0.01) showed significant differences among intervention group students during post intervention period. Moreover, students in the intervention group were more likely to use condoms during post intervention period compared to students of the control group [AOR = 4.73 (95 % CI (1.40–16.0)].

**Conclusion:**

Despite short follow up period, students in the intervention group demonstrated positive changes in HIV related comprehensive knowledge and showed better interest to go for HIV testing in the near future. Furthermore, positive changes on risky sexual behaviors were reported from the intervention group. Implementing secondary school targeted peer education by allocating appropriate amounts of resources (money, man power, materials and time) could play significant role to prevent and control HIV/AIDS among school youth.

## Background

A systematic review conducted on studies from various sub-Saharan African countries shows that both out of school and in school adolescents and youth experience risky sexual behaviors [1].

The level of comprehensive knowledge on HIV/AIDS among young people in Sub Saharan Africa remains low (36 %) for young men and (28 %) for young women) [2]. Even though youth are knowledgeable about AIDS prevention measures, many of them do little to prevent it or other sexually transmitted diseases [1].

A study done in Ethiopia on youth revealed that among those who started sexual intercourse with their boy/girl friends, only 58.5 % used condom and only 32.6 % were tested for HIV [3].

According to the Health Impact Evaluation conducted in Ethiopia in 2008, among women aged 15–24 years, those who reported consistent condom use were 48 %, limited sexual intercourse with one uninfected partner were 50 % and abstained from sex as means of preventing HIV infection were 58 % [4].

School-based HIV/AIDS health education may be an effective way to prevent the spread of HIV among adolescents and youth [5, 6]. It has been a cornerstone of youth-focused HIV prevention efforts since the early 1990s [5].

In order to maintain healthy sexual behaviors, change of risky sexual behaviors, and modify norms, peer-led HIV intervention that involve members of a specific at-risk group are thought to be more effective [7]. Compared to professional health care providers, using peer educators is less expensive [7, 8]. In addition, peer-based interventions have become a common method to effect important health-related behavior changes and it is one of the most widely used strategies to address the HIV/AIDS pandemic [6, 9].

Peer education is a strategy in which individuals from a target group provide information, training, or resources to their peers. The groups can be determined by social or demographic characteristics (e.g., age, education, type of work) or by risk-taking behavior [10]. It has been a popular method of health education for HIV prevention since 1980s, perhaps because of the positive interaction it brings between peers [11, 12]. Generally, peer education is a low cost and widely used intervention for HIV prevention especially among young population.

A systematic review and meta-analysis of peer education interventions demonstrated that peer education interventions were significantly associated with increased HIV knowledge, reduced equipment sharing among injection drug users and increased condom use [6]. But it had a non-significant effect on biological outcomes like STI [6].

A study showed that participants of school-based intervention group reported higher levels of HIV related knowledge, better condom use and more positive attitudes towards condoms at follow-up than participants in control schools [13].

The findings of a study on peer education indicated that peer education increases the knowledge of HIV/AIDS prevention methods among secondary school students [14]. Nevertheless, the findings of a systematic review have reported the limited effectiveness of peer education intervention in increasing knowledge, changing attitudes and reducing risky sexual behavior [1]. Moreover, the results of another systematic review on school based intervention programs suggested that knowledge and attitudes are easiest to change, where as behaviors are much more challenging [15].

A Meta-analysis indicated that peer education programs in developing countries are moderately effective in improving behavioral outcomes, but showed no significant impact on biological outcomes [16]. Although there are evidences for peer education intervention to bring positive changes in risky sexual behavior, the findings are not consistent among various study groups.

In general, the goal of peer education is to develop knowledge, attitudes, beliefs, and skills needed to engage in healthy behaviors [17]. However, despite the fact that peer education intervention is implemented as one of the HIV prevention and control strategies among school youth in Ethiopia including the study area, the Addis Ababa City Administration, there was no study done on its effects among various population groups in the country.

Therefore, the main purpose of this quasi-experimental study was to evaluate if peer education is an effective method of HIV prevention in high school settings. The initial hypothesis was whether the use of the specific behavioral intervention in secondary schools to prevent and control HIV/AIDS epidemic could change the knowledge, attitudes and practices of school youth in urban Ethiopia.

## Materials and methods

### Study setting and design

Secondary school based quasi-experimental study was conducted from March to June, 2013 in Addis Ababa, the Capital City of Ethiopia. According to the national census of 2007, the projected population of Addis Ababa for the year 2012 was 3,048,631 and among those, 52 .4 % were female [18, 19]. The Capital City is administratively divided into ten sub-cities (Kifle- Ketemas) and one –hundred and sixteen districts/Woredas [20]. There were 745 Primary and 163 Secondary schools (both government and non-government) in Addis Ababa. The number of students enrolled in the year 2012/2013 were 503,877(54.9 % female) and 136,636(46.1 % male) in primary and secondary schools respectively [21].

### Sampling procedure

Intervention and control groups assigned purposively. Students included in both the intervention and control groups were from grade 11 of purposely selected four secondary schools at different Sub-cities in the study area. This study was aimed at addressing one of the five different, but y interrelated specific objectives of a PhD project, with a wider scope.

Multi-stage and multiphase sampling techniques were done. In phase I: 103 public schools (76 randomly selected primary schools and all 27 secondary schools) were participated in order to address specific objective I, i.e., among 220 public schools which satisfied the inclusion and exclusion criteria about 50 % were selected based on available resources). Then in phase II, 30 public schools (15 primary and the other 15 secondary) were selected using systematic sampling techniques, in order to address specific objectives II, III&IV. Finally, in Phase III, four secondary schools (two for the intervention and the other two for control groups) among the above 27 public secondary schools were selected purposely in order to address specific objective V, i.e., the current study which is the effects of peer education intervention on HIV related sexual behaviors among secondary school youth. The detail of the methodology is described elsewhere.

This quasi–experimental study aimed at peer education intervention, and used consistent condom utilization among students as a major outcome variable. In addition, to get an adequate sample size for both the intervention and control groups, two population proportion sample size calculation formula, with 5 % type I error and 80 % power was used. Accordingly, n1 has represented students selected for the intervention group and n2 represented students selected for the control group. Moreover, for the prevalence of condom use among intervention group during post intervention period (P1), 0.88 was taken from a similar study conducted in Nigeria [22]. For the prevalence of condom use among the comparison or control group during post intervention (P2), 0.52 was taken from the same study [22]. Thus, r or ratio of n2, n1 = 1 and P = 0.88 + 0.52÷2 = 0.7. Finally, 15 % contingency was added for possible refusal to participate in the study and also to maximize the sample size as much as possible for better validity. As a result, the calculated sample size was 244 + 36 = 280 students from two secondary schools for the intervention group and another 280 students for the control group from other two purposively selected secondary schools.

Moreover, as the required number of students from each purposely selected four schools was 140, among 15–20 sections of Grade 11 in each school, 3–4 sections were randomly selected proportionately to their student population, and then 30–40 students who satisfied the inclusion criteria and also were volunteer to participate were involved in this study from each section.

### Data collection and quality control

Data were collected from the intervention and control group students using self-administered and similar questionnaire during both pre and post intervention periods. The questionnaire had several sections with various socio-demographic and sexual behaviors related variables. The questionnaire was initially prepared in English language and translated to Amharic and then back to English to maintain its consistencies in meanings and senses, by two individuals with expert knowledge in both Amharic and English languages. The questionnaire was pre- tested in two secondary schools which were not selected for the actual data collection. Then, the required corrections in language and content were done for better clarity and more understandability. The pre and post intervention data collections were facilitated by six diploma holder nurses under the supervisions of two senior health professionals (a health officer and a BSC Nurse with long work experience and were also instructors at different private Health colleges in Addis Ababa, Ethiopia and the principal investigator.

The peer education facilitators were students nominated by their peers based on their observed active class participations and good communications with students in the respective classrooms. They were also volunteers to participate in the peer education training. Two days of training was given for 30 students (15 proportionally combined male and female students or peer education facilitators from each intervention targeted secondary school). The training was given by the principal investigator and an expert from the Addis Ababa City Administration HIV/AIDS Prevention and Control Office. Furthermore, supplementary and relevant reference materials were brought from the HIV/AIDS Prevention and Control Office of the City Administration and distributed to the peer education facilitators after the training.

During the training, various topics related to the structure and functions of human reproductive organs, HIV/AIDS, prevention methods of HIV and risky sexual behaviors among in school youth, etc. were covered. The education facilitators educated their peers then after. The number of sessions conducted by each group were two times a week either after the regular school hours or using free periods as deemed necessary. Each session lasted for at least 40 min.

Throughout the intervention period, supportive supervisions were done by the principal investigator in collaboration with the respective directors and/or deputy directors of the two secondary schools selected for intervention group in order to monitor the effectiveness of the peer education program. The post intervention data were collected three months after the pre-intervention survey (from March to June, 2013).

### Ethical considerations

An ethical clearance was secured prior to the study from the Institutional Review Board of the College of Health Sciences of the Addis Ababa University. Official letter of cooperation was written from the School of Public Health of the Addis Ababa University to the Addis Ababa City Administration Education Bureau, and then similar letters were written from the Education Bureau to all concerned bodies.

Verbal consents from all participants were obtained after being fully informed about the objectives and procedures of the study. Confidentiality and anonymity were maintained. No name or other identifying information was included in the data collection instruments.

The post intervention period data were collected after three months of the base line survey.

### Data analysis

The data collected from both the intervention and control group students during pre and post intervention periods were entered first into Epi-Info software version 3.5.4 for cleaning and then exported to SPSS software version 20.0 for analysis. Descriptive statistics, such as frequencies and proportions were used to describe the study population in relation to the relevant variables. Cross tabulation of variables were also done. Dependent variables measured were comprehensive knowledge of HIV/AIDS, ever initiated sexual intercourse, ever being tested for HIV, number of sexual partners, frequency of condom use and willingness to go for HIV counseling and testing within 2 months after the survey.

The independent variables were study allocations (being in intervention or control group) and some socio-demographic variables. In order to evaluate the association between peer education and HIV/AIDS related sexual behaviors and assess for any group difference, Chi-square test was used. Furthermore, multivariable logistic regression analyses were also done in order to assess the relations among various HIV related behavioral outcome variables and characteristics of the study participants being either in peer education intervention or control group by controlling for some socio-demographic confounders like sex, age, religion and ethnicity (Table 3). The outcome variables selected were knowledge of HIV/AIDS, consistent use of condom in the previous 12 months, i.e., in the year preceding the survey and willingness to go for HIV Counseling and Testing(HCT). During chi-square tests, p-values less than 0.05 were considered as statistically significant. Furthermore, in multivariable binary logistic regression analysis, the value of an adjusted odds ratio (AOR) along with the corresponding 95 % confidence interval was used to assess the strength of the association.

### Measures of the study

The variables used for multivariate analysis of this study were selected based on their statistical significances during bivariate analyses and on their theoretical background. Moreover, some variables like comprehensive knowledge of HIV/AIDS were measured using Likert scale. Ten HIV/AIDS related knowledge questions like, heard or not about the disease HIV/AIDS, identifying major routes of HIV transmission, identifying main methods of HIV prevention, whether a healthy looking person can be infected with HIV or not, whether AIDS can be cured or not, etc. were included in the questionnaire. During the analysis stage, if a study subject responded for more than five questions correctly, she or he was considered as having comprehensive HIV/AIDS related knowledge. In addition, consistent use of condom was measured based on the study subjects responses to the question asked on the history of using condom during every act of penetrative sexual intercourse in previous 12 months of this survey. If the study subject responded as s/he used condom always in previous 12 months, it was taken as consistent use of condom to prevent the risk of HIV.

## Results

All 280 eligible study subjects of the intervention group completed the self administered anonymous questionnaires during both the pre-intervention and post intervention periods (Table 1). Nevertheless, among the control group students, those who completed the questionnaire were 280 (100 %) during the pre intervention period and 260 (92.9 %) during the post intervention period. In addition, when the averages of pre and post intervention data of each group were analyzed, 84.3 % of the intervention group and 81.6 % of the control group students were in the age group of 15–18 years (Table 1).

**Table 1:** Socio-demographic Characteristics of the study participants from secondary schools in Addis Ababa, Ethiopia, March-June 2013

Variables	Control group		Intervention group	
	Pre intervention n(%)	Post intervention n(%)	Pre intervention n(%)	Post intervention n(%)
Age group (in completed years)				
15–18	234(83.6)	223(79.6)	240(85.7)	232(82.9)
>18	46(16.4)	36(12.9)	40(14.3)	48(17.1)
Sex				
Male	99(35.4)	95(33.9)	108(38.6)	105(37.5)
Female	181(64.6)	165(58.9)	172(61.4)	175(62.5)
Marital status				
Single	266(95)	247(95)	271(96.8)	266(95)
Married and others	14(5)	11(4)	7(2.5)	13(4.6)
Religion				
Orthodox	214(76.4)	211(81.2)	199(71.1)	203(72.5)
Protestant	32.(11.4)	25(9.6)	17(6.1)	22(7.9)
Catholic	-	-	1(0.4)	1(0.4)
Muslim	30(10.7)	19(7.3)	61(21.8)	52(18.6)
Others	4(1.4)	5(1.9)	2(0.7)	2(0.7)
Ethnicity				
Amhara	114(40.7)	104(37.1)	95(33.3)	98(32)
Oromo	65(23.2)	71(25.4)	48(17.1)	46(16.4)
Tigrie	43(15.4)	42(15)	32(11.4)	36(12.9)
Ghuragie	40(14.3)	30(10.7)	79(28.2)	76(27.1)
Others	18(6.4)	13(4.6)	26(7.3)	24(8.6)

As the averages of pre and post intervention data showed, females constituted 62.0 % of the intervention group and 61.8 % of the control group students. In addition, 95.9 % of the study participants of the intervention and 95.0 % of the control group were single. Among the intervention group students, 71.8 % were Orthodox Christian Religion followers and 32.7 % were Amhara by ethnicity. Whereas, among those students in the control group, Orthodox Christian followers constituted 78.8 % and those Amhara by ethnicity were 38.9 % (Table 1).

Table 2 depicts the comparison between base line and end line findings of both the intervention and control groups in relation to selected sexual behavior indicators. The proportions of study participants in the intervention group with comprehensive HIV/AIDS related knowledge were 72.0 % during pre intervention and 82.0 % during post intervention periods (*P*-value =0.004). Among the participants of the intervention group, 44.7 % during pre intervention and 59.6 % during post intervention periods responded as they have willingness to go for HIV counseling and testing within two months after the survey (*P*-value 0.01). Thus, HIV/AIDS related comprehensive knowledge and willingness to go for HCT within two months after the survey have showed statistically significant associations with peer education intervention (Table 2).

**Table 2:** The sexual behaviors of students in the intervention and control groups during pre and post intervention period among secondary school students in Addis Ababa, Ethiopia, March-June 2013

Indicators	Control group			Intervention group		
	Base line (*N* = 280)	End line (*N* = 260)		Base line (*N* = 280)	End line (*N* = 280)	
	n(%)	n(%)	*P*-Value	n(%)	n(%)	*P*-Value
High knowledge of HIV/AIDS	218(77.9)	202(78.0)	0.97	201(72.0)	230(82.1)	0.004
Ever had initiated sexual intercourse	47(16.8)	54(20.8)	0.24	59(21.1)	62(22.3)	0.72
Ever tested for HIV	127(45.5)	125(48.3)	0.52	130(46.4)	136(49.1)	0.53
Limiting sexual partner only to one in the last12 months	14(40.0)	16(43.2)	0.78	16(34.8)	21(47.7)	0.21
Consistent use of Condom in the last 12 months	19(54.3)	10(31.2)	0.06	18(40.9)	23(53.5)	0.24
Willingness to HCT within 2 months after the survey	65(42.8)	73(54.5)	0.05	63(44.7)	84(59.6)	0.01

In addition, among the study subjects in the intervention group, as the post intervention findings indicated, 22.3 % had ever initiated sex (*P*-value 0.72), 49.1 % ever tested for HIV (*P*-value 0.53), 47.7 % reported that they had only one sexual partner (*P*-values 0.21) and 53.5 % reported consistent use of condom (*P*-values 0.24) during the year preceded the survey (Table 2).

Furthermore, the post intervention period findings of the control group didn’t show statistically significant changes between the base line and end line findings regarding HIV/AIDS related risk behaviors among secondary school students of the group. For instance, of the students of the control group, those with high knowledge of HIV/AIDS were 78.0 % (*P*-value 0.97), ever initiated sex were 20.8 % (*P*-value 0.24), ever being tested for HIV were 48.3 % (*P*-value 0.52), had only one sexual partner in previous year were 43.2 % (*P*-values 0.78), used condoms consistently in the year preceding the post intervention survey were 48.6 % (*P*-value 0.09), and showed willingness to go for HCT service within two months after the study period were 54.5 % (*P*-value 0.05) (Table 2).

In the multivariable binary logistic regression analysis being in peer education intervention group [AOR = 4.73 (95 % CI: 1.40–16.0)] and Oromo by ethnicity [AOR = 0.45 (95 % CI: 0.23–0.89)], have shown statistically significant associations with some risky sexual behavior related factors. However, comparing with the students of the control group, the intervention did not show statistically significant effect in improvising HIV related knowledge [AOR = 1.20 (95 % CI: 0.77–1.87)] and in improving the proportion of students willing to go for HCT in the near future [AOR = 1.23 (95 % CI: 0.75–2.02)] during the multivariable logistic regression analysis (Table 3).

**Table 3:** The effects of Peer education on sexual behavior of Secondary school students in the study group during post intervention period; Addis Ababa, Ethiopia, 2013

		Knowledge of HIV/AIDS			Consistent condom use in the previous 12 months			Willingness to go for HCT		
		High	Low	AOR 95 % CI	Always	Not always	AOR 95 % CI	Willing	Not Willing	AOR 95 % CI
Factors										
Group	Intervention	230	50	1.20(0.77.1.87)	23	18	4.73(1.40-16.0)	84	57	1.23(0.75-2.02)
	Control	202	57	1	10	22	1	73	61	1
Sex	Male	168	31	1.57(0.98–2.50)	15	25	2.63(0.84–8.23)	66	45	1.12(0.67–1.82)
	Female	264	76	1	18	15	1	91	73	1
Age	15–18	363	91	0.91(0.50–1.69)	20	29	2.09(0.66–6.60)	138	108	0.70(0.31–1.59)
	>18	69	15	1	13	11	1	19	10	1
Religion	Orthodox	329	84	0.71(033–1.53)	27	34	3.51(0.48–25.51)	116	82	1.0(0.50–2.0)
	Protestant, Catholic and others	42	13	0.68(026–1.77)	1	4	5.64(0.30–104.70)	12	16	0.53(0.20–1.45)
	Muslim	61	10	1	5	2	1	29	20	1
Ethnic Group	Amhara	164	38	0.76(0.40–1.45)	17	14	1.12(0.30–4.24)	54	45	0.72(0.37–1.39)
	Oromo	85	32	0.45(0.23–0.89)	1	10	0.07(0.01–0.88)	33	22	1.0(0.46–2.15)
	Ghuragie	84	21	0.59(0.28–1.23)	5	7	3.37(0.55–20.85)	31	27	0.69(0.32–1.46)
	Tigrie and others	99	16	1	10	9	1.	39	24	1

## Discussions

According to this study, the level of comprehensive knowledge on the major routes of HIV transmission and ways of prevention amongst both the study group and control group have already reached to an encouraging level during the pre-intervention period. This finding is higher than the recent Global report by UNAIDS on comprehensive HIV/AIDS knowledge among young people in sub –Saharan countries [2].

As the finding of the chi-square tests that compared the changes between the base line and end line data of both the study and control groups showed, the HIV/AIDS related knowledge of the intervention group increased significantly during the post intervention period (Table 2). This could be explained by the assumption that peer educators are the trustworthy sources of information on HIV/AIDS for the school youth. This finding is in line with similar studies conducted on the effects of peer education in various countries [5, 6, 10–12].

Although the number of students with high HIV/AIDS related knowledge increased during the post intervention period, it lost statistical significance during multivariable logistic analysis. Moreover, despite increased knowledge during post intervention period among intervention group students, risky sexual behaviors like limiting number of sexual partners only to one in the year preceding the study didn’t show statistically significant association to peer education intervention. But the findings are in agreement with some other studies conducted previously [1, 15, 16], while they are in disagreement with findings of other studies [6, 7, 13]. These observations may be explained by the assumption that the school adolescents and youths perhaps were not fully convinced with the information given by their peers on HIV related risky sexual behaviors.

Furthermore, although most of the findings of the current study did not show statistically significant differences between the intervention and control groups, peer education intervention seemed to empower students to adopt less risky sexual behaviors. These differences could be explained in terms of the current theoretical knowledge on HIV/AIDS and *P*-values < 0.05. In addition, the observed slight differences between the students in the intervention and control groups are in line with findings documented previously in various studies conducted on the effects of peer education interventions [6, 15].

In spite of positive improvements in minimizing some of the HIV related risky sexual behaviors among students involved in the study, almost equal number of the intervention and control groups did not undertake HIV counseling and testing following post intervention.

Nevertheless, significantly more students in the intervention group showed their willingness to go for HIV counseling and testing within two months after the study compared to controls. Our findings are consistent with findings of previous studies from various developing countries [3, 6, 9, 16, 17].

Nevertheless, results from the multivariable logistic regression analysis didn’t show statistically significant differences between the intervention and control groups regarding willingness to go for HCT in the near future, during the post intervention period (Table 3).

The shorter follow–up period for peer education intervention, owing to the very nature of school programs and scarcity of required resources, like free time and money, and lack of motivation among some of the peer educators and study participants due to our inability to respond positively for their request to be paid for their transportation and refreshments can be considered as the limitations of this study. In addition, information on HIV/AIDS that the students might have got from other sources like, mass media and web sites could have also an influence on the findings of this study.

## Conclusion

This study endeavored to provide some insights on the effects of peer education intervention on HIV/AIDS related sexual behaviors among secondary school youth.

Regardless of the very short follow-up period for the effect evaluation, due to the very features of the school environment and the critical financial constraints, over all, more positive sexual behavior related changes were noted among the intervention group students.

Thus, implementing peer education program in secondary schools by allocating reasonable resources could play significant role to bring about positive changes in the sexual behaviors of school youth and prevent them from the deadly epidemic, HIV/AIDS.
